# Non-Cytotoxic Nanomaterials Enhance Antimicrobial Activities of Cefmetazole against Multidrug-Resistant *Neisseria gonorrhoeae*

**DOI:** 10.1371/journal.pone.0064794

**Published:** 2013-05-21

**Authors:** Lan-Hui Li, Muh-Yong Yen, Chao-Chi Ho, Ping Wu, Chien-Chun Wang, Pawan Kumar Maurya, Pai-Shan Chen, Wei Chen, Wan-Yu Hsieh, Huei-Wen Chen

**Affiliations:** 1 Graduate Institute of Toxicology, College of Medicine, National Taiwan University, Taipei, Taiwan; 2 Kunming Branch, Taipei City Hospital, Taipei, Taiwan; 3 Department of Medicine, National Yang-Ming University, Taipei, Taiwan; 4 Department of Internal Medicine, National Taiwan University Hospital and National Taiwan University Medical College, Taipei, Taiwan; 5 Department and Institute of Pharmacology, School of Medicine, National Yang-Ming University, Taipei, Taiwan; 6 Amity Institute of Biotechnology, Amity University, Lucknow, Uttar Pradesh, India; 7 Department and Graduate Institute of Forensic Medicine, College of Medicine, National Taiwan University, Taipei, Taiwan; 8 Department of Chemistry, National Taiwan University, Taipei, Taiwan; George Mason University, United States of America

## Abstract

The emergence and spread of antibiotic-resistant *Neisseria gonorrhoeae* has led to difficulties in treating patients, and novel strategies to prevent and treat this infection are urgently needed. Here, we examined 21 different nanomaterials for their potential activity against *N. gonorrhoeae* (ATCC 49226). Silver nanoparticles (Ag NPs, 120 nm) showed the greatest potency for reducing *N. gonorrhoeae* colony formation (MIC: 12.5 µg/ml) and possessed the dominant influence on the antibacterial activity with their properties of the nanoparticles within a concentration range that did not induce cytotoxicity in human fibroblasts or epithelial cells. Electron microscopy revealed that the Ag NPs significantly reduced bacterial cell membrane integrity. Furthermore, the use of clinical isolates of multidrug-resistant *N. gonorrhoeae* showed that combined treatment with 120 nm Ag NPs and cefmetazole produced additive effects. This is the first report to screen the effectiveness of nanomaterials against *N. gonorrhoeae*, and our results indicate that 120 nm Ag NPs deliver low levels of toxicity to human epithelial cells and could be used as an adjuvant with antibiotic therapy, either for topical use or as a coating for biomaterials, to prevent or treat multidrug-resistant *N. gonorrhoeae*.

## Introduction

Although gonorrhea has been long known as a sexually transmitted disease caused by *Neisseria gonorrhoeae,* it remains an important clinical and public health problem that affects more than 62 million people worldwide annually [1]. Infection with *N. gonorrhoeae* can cause cervicitis, urethritis, proctitis, and other pelvic inflammatory diseases, leading to infertility, ectopic pregnancy, chronic pelvic pain, and potentially an increased susceptibility to HIV infection [2]. According to the treatment guidelines recommended by the Centers of Disease Control and Prevention (CDC) in 2006, cephalosporin and flouroquinolone were the two priority drugs for uncomplicated gonorrhea [3]. However, consistent worldwide increases in the resistance of gonorrhea to fluoroquinolone have made cephalosporine the only recommended drug for treatment since 2010 [4]. However, cefixime-resistant *N. gonorrhoeae* has now been reported in Japan and threatens effective disease control [5]. In addition, the broad use of antibiotics and inherent genetic mutations contribute to drug resistance [6]–[7]. Due to the reduction in treatment options for *N. gonorrhoeae*, alternative anti-microbial materials are urgently needed to maintain control of gonorrhea infections.

Nanomaterials with unique physical and chemical properties, including a small size and a high surface area-to-volume ratio, allow for an increased ability to surpass most physiological barriers to reach therapeutic targets and to interact with pathogen membranes and cell walls [8]. Hence, several nanomaterials have shown anti-microbial activity against *E. coli*, *P. aeruginosa*, *B. subtilis*, *S. aureus,* and *C. albicans* through multiple mechanisms, including the interruption of transmembrane electron transfer, disruption of the cell envelope, oxidization of cell components, or production of reactive oxygen species (ROS) [9], [10]. Nanomaterials such as silver, chitosan, and ultrafine-TiO_2_ have been used for many applications in diverse consumer products, including cosmetics, food preservation, agriculture, medical applications, and anti-microbial agents [9]. However, the effects of nanomaterials on sexually transmitted pathogens, such as *N. gonorrhoeae,* and whether they can enhance suboptimal antibiotic therapies have not been established.

In this study, we assessed the potential anti-bacterial activities of different nanomaterials against *N. gonorrhoeae*. We found that multiple-walled carbon nanotubes (MWCNTs), silica, zinc oxide, and silver nanoparticles (Ag NPs) demonstrated significant anti-bacterial activity. Specifically, the 120 nm Ag NPs were the most effective and dominant on the antibacterial activity with their properties of the nanoparticles; likely due to a loss of bacterial cell membrane integrity in the ATCC standard strain and clinical isolates. Importantly, these effects were observed at dosages that did not induce cytotoxic effects on human epithelial cells or fibroblasts. Additionally, our data revealed that Ag NPs formed complexes with cefmetazole to produce an additive effect against antibiotic-resistant clinical isolates. Thus, Ag NPs could represent a potential adjuvant for use in combination with antibiotics as a treatment for *N. gonorrhoeae*.

## Materials and Methods

### Nanomaterials and chemicals

Three different sizes of silver particles were used in this study and marked via their average diameter, 1 µm (0.9±0.4 µm, range from 0.6 to 2.2 µm measured by TEM, Sigma #327085, 2–3.5 µm manufacture announced), 120 nm (124.6±22.7 nm, range from 83.3 to 180.0 nm measured by TEM, Sigma #484059, <150 nm manufacture announced), 20 nm (20.7±13.8 nm, range from 11.4 to 62.5 nm measured by TEM, Alfa Aesar #45509, 20–40 nm manufacture announced) were purchased from Sigma (St. Louis, USA) and Alfa Aesar (Haverhill, USA). These silver particles were described by their average size in this study. Tin oxide and zinc oxide were purchased from Alfa Aesar (Haverhill, USA). Carbon black NPs were purchased from Uni Bio-Tech (Seoul, South Korea). Modified multi-walled carbon nanotubes were purchased from Nanostructured & Amorphous Materials, Inc. (Los Alamos, USA). Titanium dioxide NPs were purchased from IoLiTec Inc. (Alabama, USA). Silver nitrate was purchased from Merck (Darmstadt, Germany); cefmetazole, pluronic F-68 and the other NPs were purchased from Sigma (St. Louis, USA). The bacterial culture media, such as GC agar plates and tryptic soy broth, were purchased from Creative Media Products (Taipei, Taiwan). The suspensions of all different nanomaterials and antibiotics were diluted with the dispersant pluronic F-68 (PF-68) in phosphate-buffered saline (PBS, 0.02%) for final concentrations of 0–25 µg/ml.

### *N. gonorrhoeae* clinical isolates and American Type Culture Collection (ATCC) strains

A standard *N. gonorrhoeae* strain (ATCC 49226) was used as a control in the present study. In addition, five different multidrug-resistant clinical isolates (Isolates 1–5) from patients with acute gonococcal urethritis in the Taipei City Hospital Kunming Branch were isolated and identified using standard methods [11]. This research project was approved by the institutional review board of Taipei City Hospital.

### Cell lines and cell culture

Hs-68 (human foreskin fibroblasts), Beas2b (human bronchial epithelial cells), and HeLa (human cervical cancer epithelial cells) cells were purchased from the American Type Culture Collection (ATCC). OEC-M1 (human oral epidermal carcinoma established by Dr. Meng CL from a male Taiwanese patient [12]) cells were a generous gift from Dr. Shieh TM (China Medical University, Taiwan). Cells were cultured in RPMI-1640 or DMEM medium plus 10% fetal bovine serum (FBS) in cell culture dishes in an incubator set at 37°C and 5% CO_2_.

### Anti-bacterial test

*N. gonorrhoeae* cells (4×10^3^ cfu/ml) were incubated with different concentrations of NP suspensions for 30 min, followed by inoculation of the cells onto GC agar plates for 24 h at 37°C and 5% CO_2_. The number of colonies was then determined. All measurements were performed in triplicate.

### Measurement of leaching Ag^+^ ions in the silver particles suspensions by ICP-MS

The leaching Ag ions from different sizes of Ag particles at MIC concentration were detected by Inductively Coupled Plasma-Mass Spectrometer (ICP-MS) (Agilent 7500ce, Japan). In brief, 1 µm Ag particles at 500 µg/ml, 120 nm Ag NPs at 12.5 µg/ml, and 20 nm Ag NPs at 12.5 µg/ml were suspended in PF-68 and the suspensions containing free Ag^+^ ions were obtained by centrifuging at 15,000 g for 20 min at room temperature. A 1.6 ml aliquot of each supernatant was analyzed for Ag^+^ ion by ICP-MS.

### Cytotoxic analysis of Ag NPs in 4 human cancer and non-cancer cell lines

The Hs-68 (human foreskin fibroblasts), Beas2b (human bronchial epithelial cells), HeLa (human cervical cancer epithelial cells), or OEC-M1 (human oral epidermal carcinoma) cell lines (2×104 cells) were seeded in 12-well plates overnight and then treated with Ag NPs (5–100 µg/ml) for 24 h. At multiple time points, cells were trypsinized and stained with trypan blue to determine cell viability. The data were represented as the percentage of cell viability as compared to the control for three independent experiments.

### Scanning electron microscope (SEM) and transmission electron microscopy (TEM) analysis

Morphological changes to *N. gonorrhoeae* were investigated using scanning electron microscopy (SEM, JEOL JSM-6510LV) and transmission electron microscopy (TEM, JEOL JEM-1400). *N. gonorrhoeae* cells (1×10^7^ cfu/ml) were treated with a 5 µg/ml Ag NP suspension for 1 h, followed by centrifugation at 6,000 rpm and 4°C and fixation with 2% glutaraldehyde and 1% osmium tetroxide. Next, the cells were dehydrated with sequential treatments in 30, 50, 70, 80, 90, and 100% ethanol for 15 min. A total of 5 µl of dehydrated cells was dropped onto a glass slide and allowed to dry at room temperature. The dried samples were sputter-coated with gold for SEM. For TEM, dehydrated cells were infiltrated with Spurr's resin and polymerized at 60°C for 24 h. The sample blocks were processed into 85-nm-thick sections and placed onto 200 mesh thin bar grids and double stained with uranyl acetate and lead citrate for 10 and 4 min, respectively.

### Determination of MICs and assessment of synergism between Ag NPs and antibiotics

The minimum inhibitory concentrations (MIC) of Ag particles of different sizes and cefmetazole required to inhibit the visible growth of *N. gonorrhoeae* were estimated using a dual titration method ranging from 6.25 to 1,000 µg/ml and 1 to 64 µg/ml, respectively. *N. gonorrhoeae* suspensions (1×10^7^ cfu/ml) were treated with Ag NPs or cefmetazole for 24 h and then inoculated onto GC agar plates for another 24 h. The MIC results were recorded following the recommendations of the NCCLS [13]. The fractional inhibitory concentration index (FICi) for the combination of Ag NPs and cefmetazole, a commonly ineffective antibiotic to *N. gonorrhoeae* due to resistance, was assessed using the checkerboard test [14]. The FICi was calculated as follows: FICi  =  MIC (Ag NPs with cefmetazole)/MIC (Ag NPs only)+MIC (cefmetazole with Ag NPs)/MIC (cefmetazole only). FICi values above 2.0 indicate antagonistic effects, values between 0.5 and 2.0 indicate additive effects, and values lower than 0.5 indicate synergistic effects.

### Interaction between Ag NPs and cefmetazole (CMZ)

Cefmetazole (0.04–25 mg/ml) was incubated with or without Ag NPs (10 mg/ml) for 24 h. In addition, cefmetazole (1 mg/ml) was incubated with or without Ag NPs (5 or 25 µg/ml) for 24 h. The samples were centrifuged at 25,000 g for 15 min, and then the supernatants were measured using a UV-VIS spectrophotometer at 300 nm (SpectraMax L, USA).

### Statistics

Significant differences were determined using Student's t-test, with *P* values<0.05 indicating statistical significance.

## Results

### Anti-microbial activity of nanomaterials against *N. gonorrhoeae*

Twenty-one different types of nanomaterials were evaluated for anti-microbial activity against *N. gonorrhoeae* (ATCC 49226). *N. gonorrhoeae* cells (4×10^3^ cfu/ml) were incubated with different nanomaterials (2 mg/ml, diluted in PF-68 in PBS) for 30 min, and the bacterial cultures were then inoculated onto GC agar plates for 24 h at 37°C. Next, colony numbers were calculated and compared to PF-68 in PBS without nanomaterials. As shown in Table 1, several nanomaterials showed anti-microbial activity against *N. gonorrhoeae*, including MWCNT (10–30 µm in length, 10–20 nm in diameter), TiO_2_ (R and A-types, 25 nm), silica (3.05 µm), and zinc oxide (40–100 nm). These NPs reduced the colony numbers by 21.4%, 27.8%, 17.6%, and 17.8%, respectively. Among these nanomaterials, Ag NPs (120 nm) showed the most potent anti-bacterial activity; the growth of *N. gonorrhoeae* was completely inhibited by Ag NPs (2 mg/ml), as evidenced by the absence of colonies on the GC agar plates (Table 1).

**Table 1:** Anti-*N*. *gonorrhoeae* activity of the different nanomaterials tested.

Nanomaterials	Survival rates (%)
Carbon black	38.5±11.3 ^a^
SWCNT – carboxylic acid, 4–5 nm	48.0±8.2 ^a^
SWCNT – 5–30 µm long, 1–2 nm diameter	53.4±17.2^ c^
SWCNT- amide– 0.7–1.0 µm long, 4–6 nm diameter	104.3±6.1
MNCNT– 1.0–10 µm long, 2–15 nm diameter	52.7±13.6^ b^
MWCNT – 10–30 µm long, 10–20 nm diameter	21.4±4.0 ^a^
MWCNT – carboxylic acid, 10–30 µm long, 10–20 nm diameter	29.9±6.0 ^a^
MWCNT – 0.5–2 µm, 10–20 nm diameter	27.1±8.1 ^a^
MWCNT – OH group, 10–30 µm long, 10–20 nm diameter	26.4±3.0 ^a^
TiO2, a-type, 25 nm	40.4±10.7 ^a^
TiO2, r+a-type, 25 nm	27.8±7.1 ^a^
Silica 3.05 µm	17.6±10.7^ a^
Iron oxide, <50 nm	37.8±3.0 ^a^
Zinc, <50 nm	48.5±8.1 ^b^
Silver, 120 nm	0^ a^
C70	59.1±17.1^ c^
C60	49.1±33.1
Tungsten <150 nm	76.5±12.5^ c^
Tin oxide 1–2 mm	52.0±3.0 ^a^
Zinc oxide 40–100 nm	17.8±7.1 ^a^
Aluminum oxide 40–50 nm	59.1±7.1^ b^

*N. gonorrhoeae* were incubated with NPs (2 mg/ml) for 30 min, followed by inoculation by spreading onto GC agar plates for 24 h at 37°C. The number of colonies was determined, and the survival rates are represented as the percentage of colony formation in comparison to the control (mean±standard deviation). Here, we used a dispersant pluronic F-68 in PBS (0.02%) without nanomaterials as the control. ^a,^ ^b^, and ^c^ indicate *P* values of<0.001,<0.01, and<0.05, respectively.

### Anti-*N. gonorrhoeae* efficacy of silver particles of different sizes

**Figure 1:**
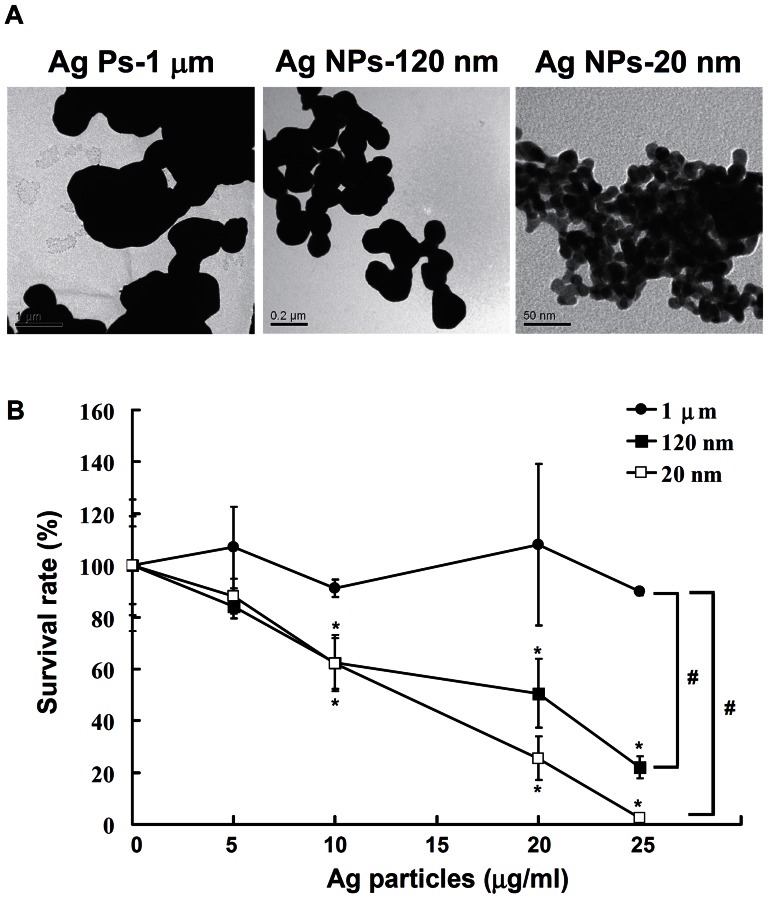
The anti-bacterial activity of Ag particles against *N. gonorrhoeae*. (A) The size and structure of cell-free Ag particle samples with dispersant were analyzed by TEM (3,500-60,000×). (B) *N. gonorrhoeae* cells (4×10^3^ cfu/ml) were incubated with Ag particles (20 nm, 120 nm, and 1 µm) at doses of 5, 10, 20, and 25 µg/ml for 30 min, followed by inoculation by spreading onto GC agar plates for 24 h at 37°C. The number of colonies was calculated, and the data are expressed as the mean±SD for three separate experiments (% of Ag NPs-free PF-68 control, * indicates *P*<0.05 compared to its' own control; ^#^ indicates *P*<0.05 compared to 1 µm Ag particles).

To investigate the size effect on their anti-bacterial activity, the MICs against an ATCC strain of *N. gonorrhoeae* for Ag particles with diameters of 1 µm, 120 nm, and 20 nm and silver nitrate were evaluated. The characterization of the Ag particles used in this study was determined by TEM (Figure 1A); which showed that these Ag particles were spherical and smooth. As shown in Table 2, the MICs of the silver nitrate and non-nanoscale Ag particles with a diameter of 1 µm were 0.5 and 500 µg/ml, whereas the MIC of the nano-scale Ag NPs (120 nm and 20 nm) was 12.5 µg/ml. Next, we examined the potential of Ag particles by conducting a colony formation assay. An Ag particle-free PF-68 solution was obtained with filtration, and its composition was analyzed. The Ag particle-free PF-68 solution had no effect on bacterial growth, and normal colony formation was observed (data not shown). To assess the effect of Ag particles on *N. gonorrhoeae*, bacterial cultures (4×10^3^ cfu/ml) were incubated with different concentrations of Ag particles (5, 10, 20, or 25 µg/ml) of different sizes (20 nm, 120 nm, and 1 µm in diameter) for 30 min, followed by inoculation by spreading onto GC agar plates for 24 h at 37°C. After incubation, the colony numbers were determined. The Ag particles exhibited dose-dependent anti-microbial activity against *N. gonorrhoeae*; the colony formation of *N. gonorrhoeae* was reduced to below 50% and 20% by incubation with 20 and 25 µg/ml, respectively, for both 120-nm- and 20-nm-diameter Ag NPs (Figure 1B). However, the 1 µm-diameter Ag particles have no significant inhibitory effects on the colony formation at a dose of 25 µg/ml. This result indicated that the Ag NPs with a diameter of 120 nm or 20 nm possessed superior anti-bacterial properties in comparison to the 1 µm Ag particles.

**Table 2:** Minimum inhibitory concentration (MIC) values of silver nanoparticles of different sizes against a standard *N. gonorrhoeae* strain.

Strain	Ag particles	MIC ( µg/ml)
*Neisseria gonorrhoeae* ATCC49226	Ag Ps (1 µm)	500
	Ag NPs (120 nm)	12.5
	Ag NPs (20 nm)	12.5
	Silver nitrate	0.5
	Cefmetazole	1

### Concentration of Ag^+^ ions leaching from Ag particles

In order to clarify if the antibacterial activity of the Ag particles is from the effects of Ag NPs-specific rather than the released Ag^+^ ions; we measured the Ag^+^ ions concentration leaching from the suspensions of different Ag particles via using ICP-MS (Table 3). At MIC dosages of different Ag particles, the Ag^+^ ions released from 1 µm, 120 nm, and 20 nm of Ag particle suspensions were 52.87, 64.57 and 42.84 ng/ml, which were less than one-tenth of the [Ag^+^] from AgNO_3_ at dose of MIC against *N. gonorrhoeae* (0.5 µg/ml), respectively. The evidence showed that the Ag^+^ ions released from Ag NPs particle size, 20 and 120 nm, were not sufficient to inhibit the growth of *N. gonorrhoeae*, which indicated that the Ag NPs may possess the dominant influence on the antibacterial activity with their properties of the nanoparticles; whereas, the free Ag^+^ ions played a limited role.

**Table 3:** Concentration of Ag^+^ ions leaching from Ag particles.

Ag particles	Total concentration ( µg/ml)	Ag^+^ ion concentration (ng/ml)
Ag Ps (1 µm)	500	52.87
Ag NPs (120 nm)	12.5	64.57
Ag NPs (20 nm)	12.5	42.84

The leaching Ag ions from different sizes of Ag particles at MIC concentration were detected by Inductively Coupled Plasma-Mass Spectrometer (ICP-MS).

### Cytotoxicity of Ag NPs of different sizes in human epithelial cells and fibroblasts

**Figure 2:**
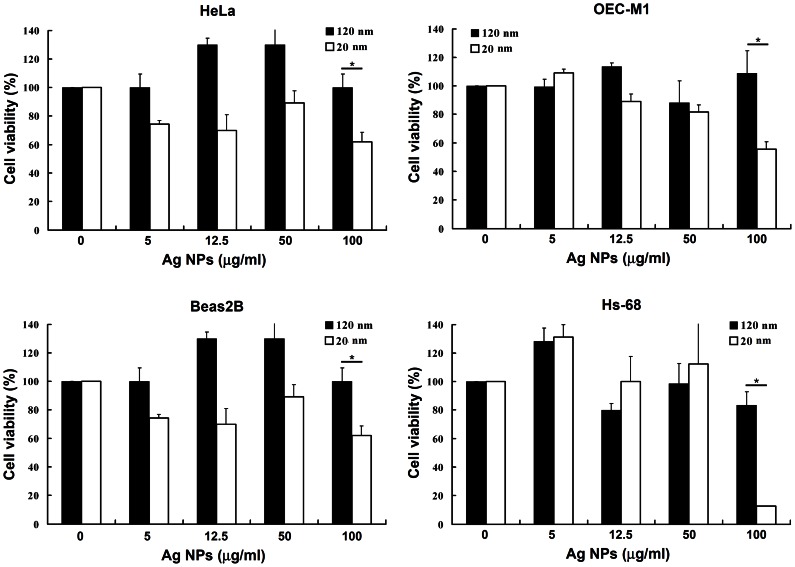
The cytotoxicity of Ag NPs of different sizes. The cytotoxicity of 120-nm and 20-nm Ag NPs against HeLa, OEC-M1, Beas2B, and Hs68 cells was evaluated by trypan blue staining (*indicates *P<*0.05).

Because the Ag NPs (20 nm and 120 nm) exhibited superior effects against *N. gonorrhoeae*, we further evaluated the cytotoxicity of Ag NPs in human epithelial and fibroblast cell lines and found that the viability of HeLa, OEC-M1, Beas2B, and Hs68 cells was not reduced by Ag NPs with a diameter of 120 nm at a dose ranging from 5 to 100 µg/ml. However, the 20-nm-diameter Ag NPs caused significant cytotoxicity at 100 µg/ml (Figure 2).

### Morphological analysis of *N. gonorrhoeae* in response to 120 nm Ag NPs

**Figure 3:**
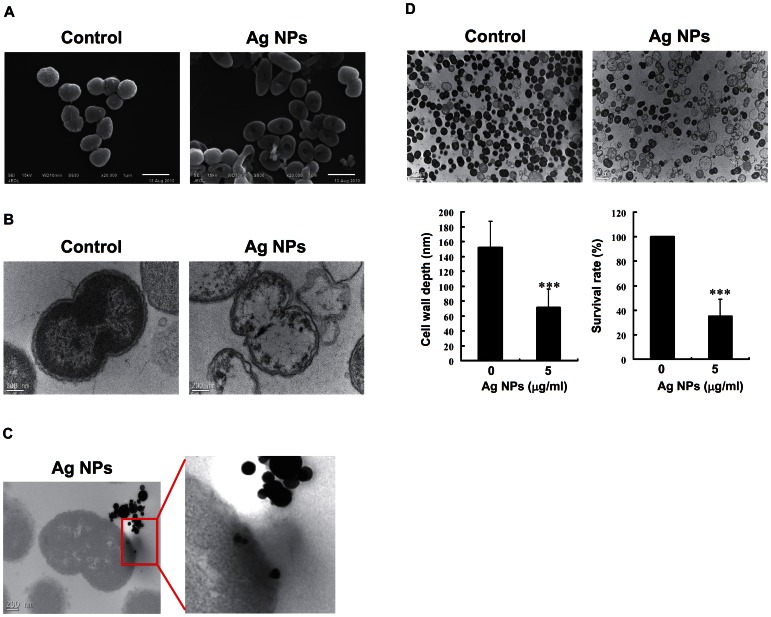
Morphological analysis of *N. gonorrhoeae* in response to 120-nm Ag NPs. *N. gonorrhoeae* cells (1×10^7^ cfu/ml) were treated with or without Ag NPs (5 µg/ml) for 1 h, and the morphology of *N. gonorrhoeae* was analyzed by (A) SEM (20,000×) and (B) TEM (13,500×). (C) *N. gonorrhoeae* cells (1×10^7^ cfu/ml) were treated with Ag NPs (10 µg/ml) for 1 h, and observations using TEM (13,500×) showed that the Ag NPs entered and accumulated in the cells. (D) The depth of the *N. gonorrhoeae* cell wall was measured by TEM (1,100×), and the cell viability was expressed as the percentage of intact bacteria in comparison to the Ag NPs-free PF-68 control. The data are expressed as the mean±SD for three separate experiments (* indicates *P*<0.001).

Based on the initial anti-*N. gonorrhoeae* activity of the 120 nm Ag NPs without cytotoxicity, we further evaluated the potential anti-bacterial effects of Ag NPs on *N. gonorrhoeae* (ATCC 49226). The bacterial morphology before and after treatment with Ag NPs was analyzed using SEM (20,000×) and TEM (1,100–13,500×) (Figure 3). *N. gonorrhoeae* cells (1×10^7^ cfu/ml) were incubated in culture media with or without the addition of 5 µg/ml Ag NPs for 1 h. The SEM images revealed that the bacterial morphology was hollow and shrank in response to treatment with Ag NPs (Figure 3A). TEM analysis was conducted to observe the intracellular morphological changes of bacterial cells after treatment with Ag NPs. Figure 3B shows the intracellular structures of control bacteria with a normal size, intact intracellular structures, and well-maintained, darkly stained intracellular contents. In contrast, the membranes of *N. gonorrhoeae* grown in culture media containing 5 µg/ml Ag NPs were clearly damaged (Figure 3B); these membranes were deformed and the intracellular structure was disorganized. Many unstained cells were also found in the *N. gonorrhoeae* cultures treated with Ag NPs, indicating that the intracellular contents had leaked out of the cells due to damage and disorganization of the cell membrane (Figure 3B). These damaged cell membranes may explain how Ag NPs entered and accumulated within the bacterial cells (Figure 3C). In addition, we examined the effect of Ag NPs on the cell wall depth of *N. gonorrhoeae* by TEM (Figure 3D). The cell wall depth of the control group was 152±35 nm, while the cell wall depth in bacteria exposed to Ag NPs was reduced to 71±25 nm. The effect of Ag NPs on cell viability was also investigated by TEM. Figure 2E shows that Ag NPs reduced the cell viability of *N. gonorrhoeae* by up to 35% as compared to the control group.

### Anti-bacterial effects of non-cytotoxic Ag NPs and cefmetazole combinations on clinical multidrug-resistant isolates of *N. gonorrhoeae*

In addition to the anti-bacterial effect of 120 nm Ag NPs against the ATCC strain of *N. gonorrhoeae*, we determined the anti-bacterial effect of these Ag NPs on clinical multidrug-resistant isolates (Table 4). The MICs of Ag NPs and cefmetazole against five resistant isolates ranged from 25 to 50 µg/ml and 8 to 16 µg/ml, respectively. The checkerboard test revealed that the combination of Ag NPs and cefmetazole exerted a greater anti-bacterial effect, as shown by a lower MIC when compared to treatment with the antibiotic alone. These data suggest that the 120 nm Ag NPs enhanced the bactericidal activity of the antibiotic. The FICs of 120 nm Ag NPs and cefmetazole were 0.25 to 1 µg/ml and 0.125 to 0.25 µg/ml, respectively, and FICi values of Ag NPs in combination with cefmetazole ranged from 0.5 to 1.25. This FICi range indicated an “additive” anti-microbial activity against the clinical multidrug-resistant isolates (Table 4).

**Table 4:** The results of a checkerboard assay using Ag NPs and cefmetazole combinations against drug-resistant *N. gonorrhoeae* clinical isolates.

Strains	Agents	MIC ( µg/ml) Alone Combination		FIC ( µg/ml)	FICi^a^	Interpretation	Parental resistance status^b^
*Neisseria gonorrhoeae* ATCC49226	Ag NPs (120 nm)	12.5	ND^d^				
	Cefmetazole	1	ND^d^				
Clinical Isolates 1	Ag NPs c	50	12.5	0.25	0.5	Additive	P, CFM,CPD, CIP,CMZ,TE
	Cefmetazole	16	4	0.25			
Clinical Isolates 2	Ag NPs c	25	12.5	0.5	0.625	Additive	P, CFM,CPD, CIP,CMZ,TE
	Cefmetazole	8	1	0.125			
Clinical Isolates 3	Ag NPs c	25	25	1	1.125	Additive	P, CIP, CMZ,TE
	Cefmetazole	8	1	0.125			
Clinical Isolates 4	Ag NPs c	25	25	1	1.125	Additive	P, CFM, CIP, CMZ,TE
	Cefmetazole	8	1	0.125			
Clinical Isolates 5	Ag NPs c	25	25	1	1.25	Additive	P, CIP, CMZ,TE
	Cefmetazole	8	2	0.25			

a FICi values above 2.0 indicate antagonistic effects, values between 0.5 and 2.0 indicate additive effects, and values lower than 0.5 indicate synergistic effects.

b Resistant status was determined using the disc diffusion method. The following abbreviations are used: penicillin (P), cefixime (CFM), cefpodoxime (CPD), ciprofloxacin (CIP), cefmetazole (CMZ), and tetracycline (TE).

c Ag NPs with a diameter of 120 nm were used in assays with clinical isolates.

d Not determined, as ATCC 49226 is sensitive to cefmetazole.

### Ag NPs can bind with cefmetazole (CMZ) and act as a drug carrier

**Figure 4:**
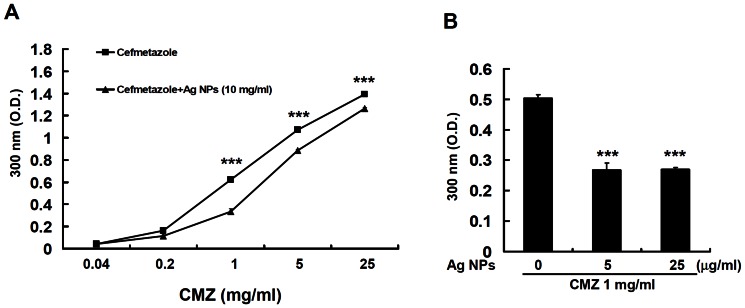
Ag NPs form a complex with cefmetazole (CMZ). (A) Cefmetazole solutions (1 to 25 mg/ml) were incubated with or without Ag NPs (10 mg/ml) for 24 hours. After centrifugation, the supernatant was analyzed at an O.D. of 300 nm using a spectrophotometer. (B) A cefmetazole solution (1 mg/ml) was incubated with or without Ag NPs (5 or 25 µg/ml) for 24 hours. After centrifugation, the supernatant was analyzed at an O.D. of 300 nm using a spectrophotometer (* indicates *P*<0.001).

The combination of Ag NPs and cefmetazole demonstrated lower MIC values when compared to the use of antibiotics alone. Therefore, we investigated whether cefmetazole and the Ag NPs could form complexes. The cefmetazole solution has an optimal absorbance at a wavelength of 300 nm, and the O.D. demonstrated a concentration-dependent increase (Figure 4A). The cefmetazole solution was incubated with Ag NPs for 24 h, and the Ag NPs were subsequently removed by centrifugation. The supernatant was analyzed using a spectrophotometer set to a wavelength of 300 nm. We found that the O.D. values of the cefmetazole solution at concentrations of 1, 5, and 25 mg/ml were significantly reduced following incubated with Ag NPs (Figure 4A). In addition, the O.D. of cefmetazole at a concentration of 1 mg/ml was significantly reduced following incubation with Ag NPs at concentrations of 5 and 25 µg/ml (Figure 4B). The decreased absorbance of the supernatant occurring after the Ag NPs and antibiotics were mixed together may suggest a more than two-fold increase in cefmetazole binding to Ag NPs. These results indicated that Ag NPs may complex with cefmetazole to deliver over twice the antibiotic dose to the bacteria, and this interaction may also abolish some of the drug-resistant mechanisms of *N. gonorrhoeae.*

## Discussion

The treatment of *N. gonorrhoeae* infections is limited because of increased antibiotic-resistant strains. As this represents a serious public health problem, it is important to develop new bactericides. In the present study, we found that Ag NPs demonstrated superior anti-microbial efficacy against *N. gonorrhoeae* among the tested nanomaterials. We also found that the MIC of cefmetazole in four clinically multidrug resistant strains was reduced to below the sensitivity threshold when combined with Ag NPs. Our data support the possible mechanisms as follows: 1) Ag NPs alone are effective in destroying the cell wall/membrane; 2) the antibacterial activity is dominated by Ag NPs-specific, whereas, the Ag^+^ ions play a limited role; 3) then, the Ag NPs and cefmetazole could contact the cells more efficiently; 4) furthermore, the Ag NPs also act as a delivery system that formed a complex and delivered more than two times the amount of antibiotics to the cells.

Ag NPs have also demonstrated toxicity against microorganisms, including multidrug-resistant bacteria like *S. aureus*, *P. aeruginosa*, *E. coli* O157: H7, and *S. pyogenes* [15]–[17]. Ag NPs also have a lower propensity to induce microbial resistance than any other material [18]. Many anti-bacterial mechanisms for Ag NPs have been proposed, including the interruption of transmembrane electron transfer [19], disruption of the cell envelope, oxidization of cell components, production of ROS [9], [10], and inhibition of cell wall, protein, and nucleic acid synthesis [15]. However, the effect of nanomaterials on *N. gonorrhoeae* remains unclear. A total of 21 nanomaterials with components of carbons, metals, metal oxides, and silica in different sizes, structures, and shapes were evaluated for anti-bacterial activity; of these, Ag NPs demonstrated the best efficiency against *N. gonorrhoeae*. In this study, all the tested NPs were dissolved in PF-68. Dos Santos et al, indicated that Ag NPs could be stabilized by dispersant PF-68 and polymers such as polyvinyl alcohol and polyvinylpyrrolidone, and the MIC value of Ag NPs might be improved by PF-68 in certain bacteria [20]; however, we found that PF-68 alone at 0.02% has no significant effect against *N. gonorrhoeae*.

**Figure 5:**
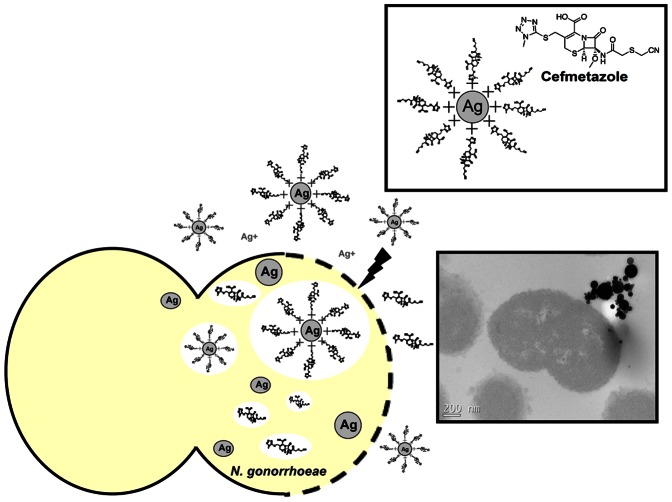
Schematic representation of the interactions between Ag NPs and cefmetazole against *N. gonorrhoeae*.

Furthermore, the results obtained by SEM and TEM showed that Ag NPs were able to attach, enter, and alter the morphology of *N. gonorrhoeae*. The Ag NPs accumulated in the bacterial membrane, likely disturbing membrane permeability and producing cell death [17]. We further hypothesized that Ag NPs may help ineffective antibiotics, such as cefmetazole, to kill *N. gonorrhoeae* by forming complexes and increasing drug entry into the cells (Figure 5). The potential mechanisms responsible for Ag NPs attachment to the *N. gonorrhoeae* membrane include electrostatic attraction [21] or interaction with sulfur-containing proteins in the bacterial membrane [22]. Furthermore, it has been reported that Ag NPs may interact with phosphorus-containing compounds, such as DNA [16].

The toxicity of Ag NPs was reported to be dependent on various factors, such as particle size [23], charge [24], and coating [25]. In the present study, Ag particles with a diameter of 1 µm showed reduced anti-bacterial activity that was 40 times lower than that of Ag NPs with a diameter of 120 nm or 20 nm. In addition to the size effect, the anti-microbial efficacy of NPs was also affected by the shape. Previous studies have shown that the anti-microbial efficacy of Ag NPs with a truncated triangular shape is superior to that of NPs with a spherical or rod shape [26]. The shape of the Ag NPs (120 nm and 20 nm) used in this study was spherical and smooth, as evidenced by TEM. Moreover, the concentration required for *N. gonorrhoeae* inhibition is compatible with previous reports [27]. In addition, we found that the leaching Ag+ ions from the Ag NPs were limited, which indicating that the antibacterial activity of the Ag NPs was dominant due to the effects of Ag NPs-specific; whereas, the Ag^+^ ions may only play a limited part. Different size particles can release different amounts of silver [28]–[29]; our data also showed that nano-size silver particles can release more Ag^+^ ions as comparing to micro-size particle. In addition, Ruden et al. suggested the concept that only micro-molar level of free Ag^+^ ions was released into solution; the free Ag^+^ions may not contribute with the bactericidal properties of the nanoparticles [30].

The use of Ag NPs in anti-bacterial or other biomedical applications is a growing field in nanotechnology; therefore, the toxicity of Ag NPs needs to be evaluated carefully. The reported cytotoxicity of Ag NPs is due to the induction of apoptosis caused by an increase in intracellular oxidative stress [21], [31], which induces inflammation [32] and damage DNA [33]. However, Ag NPs in wound dressings have been shown to promote wound healing and to reduce scar appearance in burn patients without any severe side effects except argyria [34], [35]. Hyun et al. [36] also reported that repeated inhalation of Ag NPs (13–15 nm) into the nasal respiratory mucosa of rats did not demonstrate significant toxicity. In this study, we assayed the cytotoxicity of Ag NPs in four human cancer and non-cancer cell lines and found that Ag NPs with a diameter of 120 nm were non-cytotoxic at concentrations below 100 µg/ml. Based on the formulas for spherical volume and surface area, at a concentration of 12.5 µg/ml 20-nm Ag NPs, the particle number was 216 times greater and the surface area was 36 times greater compared to the 120-nm NPs. This result indicated that smaller particles (20 nm) have a greater particle number and surface area in comparison to larger particles (120 nm) at the same mass fraction; this trend resulted in more toxicity in human cells, as shown in Figure 2. Smaller Ag NPs enter cells more easily than larger ones, and this may be the cause of the previously reported toxic effects [23], [26]–[27], [37]. These data suggest that Ag NPs with a larger size may be safe and have the potential to be used as anti-bacterial agents or products [38]. For these reasons, we focused on the bactericidal activity of Ag NPs with an average diameter of 120 nm, rather than 20 nm.

The potential applications of Ag NPs in the clinical setting included their topical use and device coating. The antibacterial activity of Ag NPs with ointments formula *in vitro* and *in vivo* has been elucidated [39]–[41]. These studies showed that Ag NPs incorporated with gel or ointments have great potential applications as an antimicrobial agent and wound healing dressing [39]–[40] and complete safety for topical application in Sprague-Dawley rats [41]. Besides topical use, they are also utilized to coat medical devices such as catheters and dentures which will tightly contact mucosae tissue [42]. We proposed that Ag NPs-coated pads and condoms maybe applicable for prophylaxis to inhibit transmitted infection [43]–[44]. Although we have tested that the 120 nm Ag NPs has no significant toxicity in several human epithelial cells under the therapeutic dosages, the potential absorption of Ag NPs via intact or damaged mucosae and the side effects from long-term usage require further assessment because genitourinary mucosae are the target of *N. gonorrhoeae* [45].

## Conclusions

The increase in multidrug-resistant *Neisseria gonorrhoeae* has a significant impact on public health, and new strategies against it are urgently needed. The antibacterial activities of nanomaterials against *N. gonorrhoeae* have not yet been reported. This is the first report to screen and demonstrate the superior effectiveness of Ag NPs against *N. gonorrhoeae* clinical isolates and ATCC strains among several types of NPs tested. We found that Ag NPs with a diameter of 120 nm showed anti-*N. gonorrhoeae* activity with an MIC at 12.5 µg/ml without inducing cytotoxicity in human epithelial cells and fibroblasts. Importantly, we found that Ag NPs-specific can effectively destroy the integrity of cell wall/membrane of *N. gonorrhoeae*, thus Ag NPs could be able to penetrate inside the bacteria and cause further damage by possibly interacting with sulfur- and phosphorus-containing proteins or DNA, and causing bacterial death. Furthermore, the leakage of cell wall/membraneare helpful for improving the delivery of cefmetazole into cells and also, Ag NPs may act as a carrier by forming complexes with cefmetazole to sensitize multidrug-resistant *N. gonorrhoeae* clinical isolates to cefmetazole treatment. These results suggest that Ag NPs could be used both as a bactericidal agent against *N. gonorrhoeae* with multidrug resistance and as a potential adjuvant combined with antibiotics for topical use in preventing or treating *N. gonorrhoeae* additively.
